# Systematic Evaluation of Genetic Variants for Polycystic Ovary Syndrome in a Chinese Population

**DOI:** 10.1371/journal.pone.0140695

**Published:** 2015-10-16

**Authors:** Yuping Xu, Zhiqiang Li, Fenglian Ai, Jianhua Chen, Qiong Xing, Ping Zhou, Zhaolian Wei, Yongyong Shi, Xiao-Jin He, Yunxia Cao

**Affiliations:** 1 Reproductive Medicine Center, Department of Obstetrics and Gynecology, The First Affiliated Hospital of Anhui Medical University, Hefei, 230022, China; 2 Institute of Reproductive Genetics, Anhui Medical University, Hefei, 230022, China; 3 Anhui Provincial Engineering Technology Research Center for Biopreservation and Artificial Organs, Hefei, 230022, China; 4 The Bio-X Institutes, Shanghai Jiao Tong University, 1954 Huashan Road, Shanghai, 200030, China; Weill Cornell Medical College Qatar, QATAR

## Abstract

To date, eleven genome-wide significant (GWS) loci (*P* < 5×10^−8^) for polycystic ovary syndrome (PCOS) have been identified through genome-wide association studies (GWAS). Some of the risk loci have been selected for replications and validated in multiple ethnicities, however, few previous studies investigated all loci. Scanning all the GWAS variants would demonstrate a more informative profile of variance they explained. Thus, we analyzed all the 17 single nucleotide polymorphisms (SNPs) mapping to the 11 GWAS loci in an independent sample set of 800 Chinese subjects with PCOS and 1110 healthy controls systematically. Variants of rs3802457 in *C9orf3* locus (*P* = 5.99×10^−4^) and rs13405728 in *LHCGR* locus (*P* = 3.73×10^−4^) were significantly associated with PCOS after the strict Bonferroni correction in our data set. The further haplotype analysis indicated that in the block of *C9orf3* gene (rs4385527 and rs3802457), GA haplotype played a protective role in PCOS (8.7 vs 5.0, *P* = 9.85×10^−6^, OR = 0.548, 95%CI = 0.418–0.717), while GG haplotype was found suffering from an extraordinarily increased risk of PCOS (73.6% vs79.2%, *P* = 3.41×10^−5^, OR = 1.394, 95%CI = 1.191–1.632). Moreover, the directions of effects for all SNPs were consistent with previous GWAS reports (*P* = 1.53×10^−5^). Polygenic score analysis demonstrated that these 17 SNPs have a significant capacity on predicting case-control status in our samples (*P* = 7.17×10^−9^), meanwhile all these gathered 17 SNPs explained about 2.40% of variance. Our findings supported that *C9orf3* and *LHCGR* loci variants were vital susceptibility of PCOS.

## Introduction

Polycystic ovary syndrome (PCOS) is a complex metabolic and endocrine disorder in reproductive-age women with a prevalence of approximately 5%-10% [1,2]. The syndrome is defined by clinical or biochemical hyperandrogenism (HA), oligomenorrhea/amenorrhea (O) and polycystic ovaries (PCO) on ultrasonography [3]. It is associated with obesity, infertility and metabolic complications including impaired glucose tolerance (IGT), insulin resistance (IR) and dyslipidemia etc. Moreover, it is also common with increased risk of endometrial cancer, type 2 diabetes (T2D) and other cardiovascular diseases [4,5,6] which leading to the detrimental impact on women's health.

Despite the pathogenesis of the disorder has not been completely elucidated yet, previous epidemiologic studies have suggested that PCOS have a strong genetic background [7]. Two genome-wide association study (GWAS) conducted in Chinese Han population indicated that common variants located in 11 genomic areas (the first GWAS: *THADA*, *LHCGR* and *DENND1A* loci; the second GWAS: *FSHR*, *C9orf3*, *INSR*, *HMGA2*, *YAP1*, *RAB5B*/*SUOX*, *TOX3* and *SUMO1P1* loci) were associated with PCOS [8,9]. And several studies in European ancestry cohorts provided further evidence for association with variants from *LHCGR*, *FSHR*, *THADA*, *YAP1* and *DENND1A* loci and PCOS [10,11,12,13]. Moreover, another recent follow-up study replicated four of the PCOS susceptibility loci (*DENND1A*, *THADA*, *FSHR* and *INSR*) in a cohort of European population, and the risk score analysis indicated the vital role in the etiology of PCOS across ethnicities for the susceptibility loci identified in the Chinese GWAS [14].

Obviously, these prior GWAS and follow-up studies have improved our understanding of the pathophysiology of PCOS. However, to our knowledge, a systematic study, to fully determine the effects of the previous reported genome-wide significant variants in an independent sample, is rarely conducted. Here, we sought to replicate association with all the genetic variants identified in the two Chinese GWAS in an independent Han Chinese cohort study, which would present a more informative profile of the previous reported variants. Additionally, a previous study suggested that some of these variants might be correlated with some phenotypes (such as hypersecretion of testosterone and luteinizing hormone, insulin resistance, and *etc*.) in PCOS patients [15]. Thus, in this study, we also investigate the correlations between the phenotypes of PCOS and the PCOS susceptibility variants.

## Materials and Methods

### Subjects

The cohort study consisted of 800 cases with PCOS and 1,110 normal controls. All the PCOS women were recruited at the Department of Obstetrics and Gynecology of the First Affiliated Hospital of Anhui Medical University, and the healthy controls data were collected from hospitals (physical examination centers) and community surveys. Medical-history and basic character profile (origin, age, height and weight) were obtained by clinical visit and surveys. All the subjected were of central Han Chinese origin. All the PCOS cases were diagnosed by at least two gynecologists according to the Revised 2003 Consensus on Diagnostic Criteria [3], and thus met any two of the following three criteria: oligo- or anovulation, clinical and/or biochemical signs of hyperandrogenism and polycystic ovary morphology. The body mass index (BMI) was calculated using the following formula: weight (kg)/height (m)^2^. For the PCOS patients, the levels of hormones containing follicular-stimulating hormone (FSH), luteinizing hormone (LH), total testosterone (T) and prolactin (PRL) were measured by chemiluminescence immunoassays. And 75-g oral glucose tolerance tests (OGTT) were conducted in the cases. Plasma glucose levels at 0 min and 2 hours after OGTT were measured using the oxidase method and insulin levels were measured by chemiluminescence immunoassays. The homeostasis model assessment of insulin resistance (HOMA-IR) was derived by the calculation: fasting plasma glucose (mmol/l) * fasting insulin (mIU/ml)/22.5. Clinical characteristics are displayed in Table 1.

**Table 1:** Characteristics of the case and the control subjects.

Characteristic	Cases(n = 800)	Controls(n = 1110)	P value
Age (ys)	26.5 ± 3.6	26.8 ± 3.8	0.082
BMI	23.5 ± 4.3	20.4 ± 2.2	<0.001
T (ng/dl)	58.2 ± 43.8	-	-
FSH (IU/L)	6.1 ± 2.2	-	-
LH (IU/L)	11.8 ± 7.1	-	-
PRL (ng/ml)	16.5 ± 8.6	-	-
Fasting Glucose (mmol/l)	5.5 ± 0.9	-	-
2h-Glucose (mmol/l)	6.8 ± 2.7	-	-
Fasting Insulin (μIU/mL)	14.0 ± 18.1	-	-
2h-Insulin (μIU/mL)	83.4 ± 69.3	-	-
HOMA-IR	3.6 ± 4.6	-	-

Data are presented as the mean ± standard deviation. BMI: body mass index; T: total testosterone; FSH: follicular-stimulating hormone; LH: luteinizing hormone; PRL: prolactin; 2h-Glucose and 2h-Insulin: glucose and insulin levels at 2 hours after OGTT; HOMA-IR: homeostasis model assessment-insulin resistance.

This study was conducted in accordance with the tenets of the Declaration of Helsinki and its Amendments Approval was received from the Ethics Committee of Anhui Medical University. After providing a complete description of the study to the subjects, written informed consent was obtained.

### Genotyping

The 17 previously reported independent single nucleotide polymorphisms (SNPs) from 11 loci were genotyped in this study. Genotyping was performed using the Ligase Detection Reaction-Polymerase Chain Reaction (LDR-PCR) method. For each plate, one sample was randomly selected to be genotyped twice, and all the non-missing genotypes were consistent. The successful rate of genotyping for all SNPs was over 95%, and the genotype distributions in both cases and controls obeyed Hardy–Weinberg equilibrium (HWE).

### Statistical analysis

HWE analysis was conducted adopting PLINK [16], and a *P* value of > 0.05 was considered obeying HWE. We analyzed the association between the 17 SNPs and PCOS using additive logistic regression model. In order to eliminate the potential effect of BMI, BMI was considered as a covariate for adjustment. The haplotype analyses of *THADA*, *FSHR*, *C9orf3*, *DENND1A* genes were also performed with SHEsis software, available online http://analysis.bio-x.cn/myAnalysis.php. The haplotypes were generated using expectation–maximization algorithm. Frequencies of the different haplotypes were compared with Chi Square analysis. Given the prior evidence of association with PCOS for the SNPs, adjustment for multiple comparisons was finished adopting Bonferroni correction. After correction, *P* value < 2.9 × 10^−3^ was considered significant. Linear regression analyses with BMI covariate were used to test association between the SNPs and PCOS phenotypes (hormones levels: T, FSH, LH and RPL; and glucose homeostasis: fasting glucose, 2-hour postprandial glucose, fasting insulin, 2-hour insulin and HOMA-IR). All phenotypes with abnormal distributions were logarithmically transformed. The analyses were also carried out using SNPTEST [17]. A Bonferroni corrected *P* value of 1.47 × 10^−3^ (0.05/34; accounting for 17 SNPs against 2 trait categories: hormones and glucose homeostasis) was considered statistically significant in genotype–phenotype analyses.

Polygenic scoring analysis was conducted as listed below: the risk-profiles of the 17 SNPs from the previous reports were selected to generate scores using PLINK “—score” function. For each individual, the sum across SNPs of the number of reference alleles (0,1 or 2) at that SNP multiplied by the score for that SNP was calculated, and then the average score per non-missing SNP was generated. The case-control status was predicted by logistic regression analysis of polygenic scores. Nagelkerke R2 showed an estimate of the variance explained. We conducted sign tests using the binomial distribution, comparing the direction of ORs of the 17 SNPs between current study and previous GWAS reporters. *P* value was generated under the null hypothesis (H0: p = 0.50).

## Results

### The Basic Information of the Study Population and the Selected SNPs

The clinical characteristics of the PCOS patients and controls have presented in Table 1. Average ages were 26.5 years in the cases and 26.0 years in the controls, respectively. Mean BMI was 23.5 in the former group, as well as 20.4 in the latter. The hormone and glucose homeostasis were only measured in the cases, and the average values for the characteristics were listed in Table 1. The basic information of the 17 SNPs detected was demonstrated in Table 2. The results indicated that all the study population obeyed the Hardy–Weinberg equilibrium.

**Table 2:** Information of the 17 SNPs detected.

Gene	SNP ref.	Loci	Allele	Location	*P*_HWE_
*THADA*	rs12468394	2p21	C>A	Intron	0.190
*THADA*	rs13429458	2p21	A>C	Intron	0.456
*THADA*	rs12478601	2p21	C>T	Intron	0.199
*LHCGR*	rs13405728	2p16.3	A>G	Intron	0.310
*FSHR*	rs2268361	2p16.3	C>T	Intron	0.191
*FSHR*	rs2349415	2p16.3	C>T	Intron	0.153
*C9orf3*	rs4385527	9q22.32	G>A	Intron	0.751
*C9orf3*	rs3802457	9q22.32	G>A	Intron	0.882
*DENND1A*	rs10818854	9q33.3	G>A	Intron	0.344
*DENND1A*	rs2479106	9q33.3	A>G	Intron	0.706
*DENND1A*	rs10986105	9q33.3	T>C	Intron	0.247
*YAP1*	rs1894116	11q22.1	A>G	Intron	0.655
*RAB5B/SUOX*	rs705702	12q13.2	A>G	Upstream	0.916
*HMGA2*	rs2272046	12q14.3	A>C	Intron	0.250
*TOX3*	rs4784165	16q12.1	T>G	Intron	0.775
*INSR*	rs2059807	19p13.3	A>G	Intron	0.825
*SUMO1P1*	rs6022786	20q13.2	G>A	intergenic region	0.825

SNP ref.: single necleaue polymorphism reference; Allel: major > minor HWE: Hardy–Weinberg equilibrium, *P*_HWE_ more than 0.05 indicated that the cases and the controls obeyed Hardy–Weinberg equilibrium.

### The Association Analysis Between the 17 SNPs Variants and PCOS

Association analysis between the 17 SNPs and PCOS were systematically illustrated in Table 3. The directions of effects for all these SNPs were consistent with previous reports (binomial test *P* = 1.53 × 10^−5^). Of the 17 SNPs, rs13405728 of *LHCGR* gene and rs3802457 of *C9orf3* gene were closely related to PCOS even after the rigorous Bonferroni correction. The G allele frequency of rs13405728 in the controls demonstrated extraordinarily higher than that of the PCOS cases (24.6% vs 19.2%, *P* = **3.73 × 10** ^**−4**^). The results also indicated that the G allele played a protective role in PCOS (OR = 0.72, 95 CI = 0.60–0.86). However, A allele frequency of rs3802457 of *C9orf3* gene in the controls showed extremely lower than PCOS cases (6.4% vs 10.1%, *P* = **5.99 × 10** ^**−4**^). Thus, it suggested that the A allele was a risk susceptibility to PCOS (OR = 0.62, 95%CI = 0.48–0.82).

**Table 3:** Association analysis of previous GWAS risk variants with PCOS, adjusted for BMI.

Gene	SNP	Minor allele	Controls N (%)	Cases N (%)	OR [95% CI]	*P* value	OR (*P* value) of the previouse GWAS study
*THADA*	rs12468394	A	541(24.4)	343(21.4)	0.79 [0.66–0.94]	9.10 × 10^−3^	**0.72 (1.59 × 10** ^**−20**^ **)**
*THADA*	rs13429458	C	348(16.4)	211(13.8)	0.78 [0.63–0.96]	0.020	**0.67 (1.73 × 10** ^**−23**^ **)**
*THADA*	rs12478601	T	574(25.9)	369(23.1)	0.80 [0.67–0.94]	8.92 × 10^−3^	**0.72 (3.48 × 10** ^**−23**^ **)**
*LHCGR*	rs13405728	G	546(24.6)	307(19.2)	0.72 [0.60–0.86]	**3.73 × 10** ^**−4**^	**0.71 (7.55 × 10** ^**−21**^ **)**
*FSHR*	rs2268361	T	1121(50.7)	734(46.3)	0.87 [0.75–1.00]	0.055	0.87 (9.89 × 10^−13^)
*FSHR*	rs2349415	T	391(18.4)	304(20.6)	1.15 [0.95–1.39]	0.147	1.19 (2.35 × 10^−12^)
*C9orf3*	rs4385527	A	251(15.8)	392(17.7)	0.86 [0.71–1.05]	0.140	0.84 (5.87 × 10^−9^)
*C9orf3*	rs3802457	A	102(6.4)	224(10.1)	0.62 [0.48–0.82]	**5.99 × 10** ^**−4**^	**0.77 (5.28 × 10** ^**−14**^ **)**
*DENND1A*	rs10818854	A	182(8.2)	153(9.6)	1.29 [1.00–1.67]	0.055	1.51 (9.40 × 10^−18^)
*DENND1A*	rs2479106	G	488(22.0)	354(22.1)	1.02 [0.86–1.21]	0.835	1.34 (8.12 × 10^−19^)
*DENND1A*	rs10986105	C	153(7.0)	145(9.3)	1.48 [1.13–1.94]	4.41 × 10^−3^	**1.47 (6.90 × 10** ^**−15**^ **)**
*YAP1*	rs1894116	G	453(20.5)	366(23.1)	1.30 [1.09–1.55]	3.79 × 10^−3^	**1.27 (1.08 × 10** ^**−22**^ **)**
*RAB5B/SUOX*	rs705702	G	530(23.9)	433(27.1)	1.18 [0.99–1.39]	0.059	1.27 (8.64 × 10^−26^)
*HMGA2*	rs2272046	C	80(4.0)	50(3.5)	0.87 [0.59–1.29]	0.499	0.70 (1.95 × 10^−21^)
*TOX3*	rs4784165	G	726(35.1)	534(36.6)	1.05 [0.90–1.23]	0.540	1.15 (3.64 × 10^−11^)
*INSR*	rs2059807	G	641(29.5)	508(32.8)	1.15 [0.98–1.35]	0.082	1.14 (1.09 × 10^−8^)
*SUMO1P1*	rs6022786	A	737(34.1)	541(35.3)	1.05 [0.90–1.22]	0.541	1.13 (1.83 × 10^−9^)

OR, odds ratio; 95% CI, 95% confidence interval; SNPs with *P* value less than 2.9 × 10^−3^ are marked in bold.

Moreover, additional four variants in the *DENND1A*, *THADA* and *YAP1* loci presented marginally significance (*P* value < 0.01). They were rs10986105 at *DENND1A* locus (OR = 1.48, 95%CI = 1.13–1.94, *P* = 4.41 × 10^−3^), rs12478601/rs12468394 at *THADA* locus (OR = 0.79 and 0.80, *P* = 8.92 × 10^−3^ and 9.10 × 10^−3^) and rs1894116 at *YAP1* locus (OR = 1.30, 95%CI = 1.09–1.55, *P* = 3.79 × 10^−3^), respectively (**Table 3**).

To assess the polygenic signal from the previous GWAS report adapted to our samples, we conducted a polygenic scoring analysis. We used the published GWAS data set as the training set, and tested on our data set. It indicates that the 17 SNPs have a significant capacity to predict case-control status in our samples (*P* = 7.17 × 10^−9^), and the proportions of variance in case-control status explained by these SNPs is 2.40% (based on all 17 SNPs analyzed together). The non-significant SNPs set (n = 15), where the significant *C9orf3* and *LHCGR* SNPs (rs13405728 and rs3802457) are not included, can explain about 1.42% of the variance (*P* = 7.93 × 10^−6^). Whereas, the SNPs with association P > 0.05 (n = 10) can explain about 0.95% of the variance (P = 2.47 × 10–4). These scores reveal association with PCOS for SNPs that individually were not powered to show association

### Haplotype Analysis of THADA, FSHR, C9orf3 and DENND1A and PCOS

In this systematic study, four blocks were produced for the further haplotype analysisme in *THADA*, *FSHR*, *C9orf3* and *DENND1A* loci. After strict Bonferroni correction, *P* value less than 0.125 was considered to be significantly different. From the results in Table 4, GA haplotype (rs4385527 and rs3802457 of *C9orf3* gene) in the controls was significantly higher than that of the cases (8.7% vs 5.0%, *P* = 9.85×10^−6^). This indicated that women with GA haplotype might be a protective factor (OR = 0.548, 95%CI = 0.418–0.717) against PCOS. However, GG haplotype in the cases was significantly higher than that in the controls (79.2% vs 73.6%, *P* = 3.41×10^−5^), which revealed GG haplotype to be a risk factor suffering from PCOS (OR = 1.394, 95%CI = 1.191–1.632).

**Table 4:** Haplotype analysis of *THADA* gene, *FSHR* gene, *C9orf3* gene and *DENND1A* gene between the controls and the cases.

Haplotype	Controls N (%)	Cases N (%)	OR[95%CI]	*P* value
*THADA* gene				
A A T	144.17(6.8)	104.97(6.9)	0.996 [0.767–1.293]	0.977
A C T	311.57(14.7)	190.78(12.5)	0.814[0.670–0.988]	0.038^#^
C A C	1571.86(73.9)	1185.74(77.5)	1.162[0.986–1.370]	0.073
*FSHR* gene				
C C	752.99(35.7)	534.04(36.4)	1.034 [0.900–1.188]	0.635
C T	296.01(14.0)	248.96(17.0)	1.255 [1.045–1.508]	0.015^#^
T C	972.01(46.0)	628.96(42.9)	0.881 [0.771–1.008]	0.065
T T	90.99(4.3)	54.04(3.7)	0.850 [0.603–1.198]	0.353
*C9orf3* gene				
A G	362.06(16.4)	229.14(14.4)	0.860 [0.719–1.030]	0.101
G A	193.07(8.7)	79.15(5.0)	0.548 [0.418–0.717]	**9.85 × 10** ^**−6**^
G G	1626.93(73.6)	1259.86(79.2)	1.394 [1.191–1.632]	**3.41 × 10** ^**−5**^
*DENND1A* gene				
A G C	143.85(6.5)	135.93(8.7)	1.351 [1.059–1.725]	0.015^#^
G A A	1683.77(76.5)	1213.31(77.6)	1.032 [0.880–1.210]	0.698
G G A	328.08(14.9)	193.61(12.4)	0.799 [0.660–0.968]	0.021^#^

The SNP sites for the block of THADA gene is: rs12468394, rs13429458 and rs12478601. The SNP sites for the block of FSHR gene is: rs2268361 and rs2349415. The SNP sites for the block of C9orf3 gene is: rs4385527 and rs3802457. The SNP sites for the block of DENND1A gene is: rs10818854, rs2479106 and rs10986105. P value less than 0.0125 are marked in bold. P value less than 0.05 but more than 0.0125 are marked with^#^

We also explored some other haplotypes presented with marginally significance between the controls and the PCOS cases (*P* values marked with^#^ in Table 4), however, the differences failed to reach statistical level after strict Bonferroni correction.

Association analyses of the 17 SNPs against quantitative traits were conducted in the PCOS group (S1 Table). Unfortunately, none of the SNPs illustrated significant association with the quantitative traits after multiple test corrections.

## Discussion

PCOS is defined as a complicated genetic disease, whereas the etiology has not been elucidated sufficiently. Previous GWAS had identified 11 loci (including 17 independent SNPs) associated with PCOS with *P* <5×10^−8^ [8,9]. McAllister et al.[18] predicted that the candidate genes by the PCOS GWAS might comprise a hierarchical signaling network which would influence the theca cell hormone biosynthesis. This would bring a completely new era of the genetic diagnostic for PCOS. In this study, we tended to confirm the effects of all these variants on PCOS adopting an independent sample. We found that the effects of all the 17 SNPs were directionally consistent with those in previous Chinese GWAS [9] and other ethnicity [12,13]. Even after strict Bonferroni correction, two of the 11 previously reported susceptibility loci demonstrated significant association with PCOS in our sample (*LHCGR* and *C9orf3*). The further polygenic score analysis showed that these SNPs collectively accounted for approximately 2.40% of the variance to PCOS, suggested an important role in the etiology of PCOS for them.

To our knowledge, it was the first systematic replication study of all the reported SNPs variants from the GWAS studies with strict statistical analysis (Bonferroni correction). The validity of the findings should be convincing as the bias was controlled to the lowest level. Additional scores for non-significant SNPs (*P*>2.9×10^−3^ or 0.05) revealed association with PCOS for SNPs that individuals were not valid to show association, whereas risk score analysis demonstrates the validity contrasted with individual SNP analysis.

In the present study, after the strict Bonferroni correction, A allele of rs3802457 (*C9orf3* gene) was considered as a risk susceptibility to PCOS (*P* = 5.99×10^−4^). Compared with the previous GWAS results, the risk rate were extremely similar to each other (OR:0.62 vs 0.77). It was well acknowledged that haplotype analysis could identify chromosomes where sequencing might be finished to explore functional variants. To our knowledge, it was the first time to perform the haplotype analysis of *C9orf3* gene (rs4385527 and rs3802457) in such a large cohort study of Chinese PCOS patients. The results revealed GG haplotype of C9orf3 gene as a risk factor suffering from PCOS, whereas haplotype GA might be a protective factor against PCOS. C9or*f3*, known as Aminopeptidase O, encodes a member of the M1 zinc aminopeptidase family that catalyzes the removal of an amino acid from the amino terminus of a protein or peptide. It has been reported to play an important role in the generation of angiotensin IV [19,20]. However, the function of *C9orf3* was rarely discussed. Kerns et al. reported that variants within *C9orf3* was detected to be associated with the development of erectile dysfunction in African-American men who have received radiotherapy for prostate cancer [21]. Arefi et al.[22] considered that rennin-angiotensin system (RAS) played a vital role in the pathogenesis of PCOS including insulin resistance. Renin activity combining with other clinical variables behaved more sensitive to diagnose women with PCOS according to the previous study [23]. Therefore, we hypothesized that *C9orf3* variants might lead to rennin-angiotensin system abnormality which would cause or deteriorate the pathogenesis of PCOS. Although rs3802457 located in intron, there were plentiful possible mechanisms that could explain the alteration of C9orf3, such as: splicing variation, microRNAs regulation or some other undetected copy-number variation (CNV), etc.

The *LHCGR* gene encodes the LH/choriogonadotropin (HCG) receptor of two structurally homologous glycoproteins which belongs to the family of G-protein coupled receptors [24]. *LHCGR* is expressed on theca cells, differentiated granulosa cells (GCs) and luteal cells of the ovary. It has been well known that, LH induces follicular development containing maturation, ovulation and luteinization during the midcycle surge of female menstrual cycle and hCG is required for the maintenance of a successful pregnancy [25,26]. *LHCGR* SNP variants were identified as PCOS susceptibility loci (rs13405728) in the first Chinese GWAS[8]. In our study population, we found that G allele frequency of rs13405728 in the controls was closely associated with the PCOS cases (24.6% vs 19.2%, *P* = 3.73 × 10^−4^) and the risk factor behaved extremely similar to the former GWAS study (OR: 0.72 vs 0.71). Meanwhile, a genotype-phenotype correlation analysis also revealed that *LHCGR* (rs13405728) variant was associated with the phenotype in PCOS with oligo-ovulation or anovulation in another Chinese population [15]. Our results combining with the previous reports provided forceful supports for the relations between *LHCGR* gene variants and PCOS in Chinese population. Interestingly, due to the rarity of rs13405728 in Europeans (comparing to Chinese), most of the previous replication analysis in European cohorts study failed to explore any significant evidence between rs13405728 and PCOS [10,11,13,27]. But one excellent mapping study in a European ancestry cohort identified another two SNPs in *LHCGR* locus were significantly associated with PCOS[12]. Nevertheless, plentiful evidences from different ethnicity have confirmed that *LHCGR* variants as a vital genetic factor for the pathogenesis of PCOS.

Although the present results offered the most significant genetic value for C9orf3 and LHCGR variants of PCOS, the OR of the two variants for association with PCOS was not particularly greater than the other variants. This raised the possibility that they might be emerged as the most significant by statistical chance, even with the current sample size.

In summary, we successfully confirmed that two (*LHCGR* and *C9orf3*) of 11 previously identified PCOS loci by a systematic variants association study in an independent Chinese cohort. Haplotype analysis of *C9orf3*gene revealed GG haplotype as a risk genetic factor for PCOS. However, the detailed mechanisms by which *LHCGR* and *C9orf3* caused PCOS need further clinical investigation and functional studies in *vitro* and in *vivo*.

## Supporting Information

S1 Table Association analysis of quantitative traits with genotype in women with PCOS, adjusted for BMI. (DOC)
