# Supplementary material for: Systematic Evaluation of Genetic Variants for Polycystic Ovary Syndrome in a Chinese Population
Source: PLoS One. 2015 Oct 16;10(10):e0140695. doi: 10.1371/journal.pone.0140695 (PMC4608705; doi:10.1371/journal.pone.0140695)
Supplement: S1 Table — (DOC) [file pone.0140695.s001.doc]

**S1 Table. Association analysis of quantitative traits with genotype in women with PCOS, adjusted for BMI**

| **Trait** | **Loci** | **Gene** | **SNP** | **Effect allele** | **P value** | **Beta** | **SE** |
| --- | --- | --- | --- | --- | --- | --- | --- |
| T | 2p21 | *THADA* | rs12468394 | C | 0.073 | 0.107 | 0.059 |
| T | 2p21 | *THADA* | rs13429458 | C | 0.344 | -0.070 | 0.074 |
| T | 2p21 | *THADA* | rs12478601 | T | 0.225 | -0.070 | 0.058 |
| T | 2p16.3 | *LHCGR* | rs13405728 | G | 0.361 | -0.059 | 0.065 |
| T | 2p16.3 | *FSHR* | rs2268361 | T | 0.908 | -0.006 | 0.049 |
| T | 2p16.3 | *FSHR* | rs2349415 | T | 0.374 | -0.060 | 0.068 |
| T | 9q22.32 | *C9orf3* | rs4385527 | G | 0.580 | 0.038 | 0.069 |
| T | 9q22.32 | *C9orf3* | rs3802457 | G | 0.123 | -0.159 | 0.103 |
| T | 9q33.3 | *DENND1A* | rs10818854 | G | 0.659 | -0.038 | 0.086 |
| T | 9q33.3 | *DENND1A* | rs2479106 | G | 0.645 | -0.028 | 0.060 |
| T | 9q33.3 | *DENND1A* | rs10986105 | C | 0.818 | 0.021 | 0.089 |
| T | 11q22.1 | *YAP1* | rs1894116 | G | 0.992 | 0.001 | 0.060 |
| T | 12q13.2 | *RAB5B/SUOX* | rs705702 | G | 0.983 | -0.001 | 0.056 |
| T | 12q14.3 | *HMGA2* | rs2272046 | C | 0.100 | 0.238 | 0.144 |
| T | 16q12.1 | *TOX3* | rs4784165 | T | 0.741 | 0.012 | 0.035 |
| T | 19p13.3 | *INSR* | rs2059807 | G | 0.669 | 0.023 | 0.055 |
| T | 20q13.2 | *SUMO1P1* | rs6022786 | G | 0.744 | 0.018 | 0.054 |
| FSH | 2p21 | *THADA* | rs12468394 | C | 0.920 | -0.006 | 0.059 |
| FSH | 2p21 | *THADA* | rs13429458 | C | 0.626 | 0.035 | 0.072 |
| FSH | 2p21 | *THADA* | rs12478601 | T | 0.782 | -0.016 | 0.058 |
| FSH | 2p16.3 | *LHCGR* | rs13405728 | G | 0.008 | -0.170 | 0.064 |
| FSH | 2p16.3 | *FSHR* | rs2268361 | T | 0.925 | 0.005 | 0.049 |
| FSH | 2p16.3 | *FSHR* | rs2349415 | T | 0.456 | 0.046 | 0.061 |
| FSH | 9q22.32 | *C9orf3* | rs4385527 | G | 0.098 | -0.113 | 0.068 |
| FSH | 9q22.32 | *C9orf3* | rs3802457 | G | 0.327 | -0.100 | 0.103 |
| FSH | 9q33.3 | *DENND1A* | rs10818854 | G | 0.574 | -0.048 | 0.086 |
| FSH | 9q33.3 | *DENND1A* | rs2479106 | G | 0.463 | 0.044 | 0.060 |
| FSH | 9q33.3 | *DENND1A* | rs10986105 | C | 0.643 | 0.041 | 0.089 |
| FSH | 11q22.1 | *YAP1* | rs1894116 | G | 0.092 | -0.101 | 0.060 |
| FSH | 12q13.2 | *RAB5B/SUOX* | rs705702 | G | 0.444 | 0.043 | 0.056 |
| FSH | 12q14.3 | *HMGA2* | rs2272046 | C | 0.089 | 0.232 | 0.137 |
| FSH | 16q12.1 | *TOX3* | rs4784165 | T | 0.070 | 0.100 | 0.055 |
| FSH | 19p13.3 | *INSR* | rs2059807 | G | 0.087 | 0.094 | 0.055 |
| FSH | 20q13.2 | *SUMO1P1* | rs6022786 | G | 0.332 | -0.049 | 0.050 |
| LH | 2p21 | *THADA* | rs12468394 | C | 0.748 | -0.019 | 0.058 |
| LH | 2p21 | *THADA* | rs13429458 | C | 0.335 | 0.067 | 0.070 |
| LH | 2p21 | *THADA* | rs12478601 | T | 0.194 | 0.073 | 0.057 |
| LH | 2p16.3 | *LHCGR* | rs13405728 | G | 0.106 | -0.102 | 0.063 |
| LH | 2p16.3 | *FSHR* | rs2268361 | T | 0.127 | -0.073 | 0.048 |
| LH | 2p16.3 | *FSHR* | rs2349415 | T | 0.415 | 0.052 | 0.064 |
| LH | 9q22.32 | *C9orf3* | rs4385527 | G | 0.901 | -0.008 | 0.067 |
| LH | 9q22.32 | *C9orf3* | rs3802457 | G | 0.849 | -0.019 | 0.100 |
| LH | 9q33.3 | *DENND1A* | rs10818854 | G | 0.687 | -0.034 | 0.084 |
| LH | 9q33.3 | *DENND1A* | rs2479106 | G | 0.147 | 0.085 | 0.058 |
| LH | 9q33.3 | *DENND1A* | rs10986105 | C | 0.560 | 0.050 | 0.087 |
| LH | 11q22.1 | *YAP1* | rs1894116 | G | 0.896 | 0.008 | 0.059 |
| LH | 12q13.2 | *RAB5B/SUOX* | rs705702 | G | 0.065 | 0.101 | 0.055 |
| LH | 12q14.3 | *HMGA2* | rs2272046 | C | 0.535 | -0.086 | 0.138 |
| LH | 16q12.1 | *TOX3* | rs4784165 | T | 0.688 | 0.022 | 0.054 |
| LH | 19p13.3 | *INSR* | rs2059807 | G | 0.846 | 0.010 | 0.053 |
| LH | 20q13.2 | *SUMO1P1* | rs6022786 | G | 0.771 | -0.015 | 0.053 |
| PRL | 2p21 | *THADA* | rs12468394 | C | 0.485 | -0.041 | 0.059 |
| PRL | 2p21 | *THADA* | rs13429458 | C | 0.723 | 0.026 | 0.073 |
| PRL | 2p21 | *THADA* | rs12478601 | T | 0.885 | 0.008 | 0.058 |
| PRL | 2p16.3 | *LHCGR* | rs13405728 | G | 0.708 | -0.024 | 0.064 |
| PRL | 2p16.3 | *FSHR* | rs2268361 | T | 0.504 | -0.033 | 0.049 |
| PRL | 2p16.3 | *FSHR* | rs2349415 | T | 0.050 | 0.130 | 0.066 |
| PRL | 9q22.32 | *C9orf3* | rs4385527 | G | 0.456 | 0.051 | 0.068 |
| PRL | 9q22.32 | *C9orf3* | rs3802457 | G | 0.723 | 0.036 | 0.103 |
| PRL | 9q33.3 | *DENND1A* | rs10818854 | G | 0.289 | 0.091 | 0.086 |
| PRL | 9q33.3 | *DENND1A* | rs2479106 | G | 0.868 | 0.010 | 0.060 |
| PRL | 9q33.3 | *DENND1A* | rs10986105 | C | 0.103 | -0.143 | 0.088 |
| PRL | 11q22.1 | *YAP1* | rs1894116 | G | 0.644 | 0.028 | 0.060 |
| PRL | 12q13.2 | *RAB5B/SUOX* | rs705702 | G | 0.268 | 0.062 | 0.056 |
| PRL | 12q14.3 | *HMGA2* | rs2272046 | C | 0.588 | 0.073 | 0.136 |
| PRL | 16q12.1 | *TOX3* | rs4784165 | T | 0.189 | 0.072 | 0.055 |
| PRL | 19p13.3 | *INSR* | rs2059807 | G | 0.628 | -0.026 | 0.055 |
| PRL | 20q13.2 | *SUMO1P1* | rs6022786 | G | 0.780 | -0.015 | 0.054 |
| Fasting Glucose | 2p21 | *THADA* | rs12468394 | C | 0.802 | 0.014 | 0.057 |
| Fasting Glucose | 2p21 | *THADA* | rs13429458 | C | 0.841 | 0.014 | 0.070 |
| Fasting Glucose | 2p21 | *THADA* | rs12478601 | T | 0.561 | -0.032 | 0.056 |
| Fasting Glucose | 2p16.3 | *LHCGR* | rs13405728 | G | 0.983 | -0.001 | 0.062 |
| Fasting Glucose | 2p16.3 | *FSHR* | rs2268361 | T | 0.159 | 0.066 | 0.047 |
| Fasting Glucose | 2p16.3 | *FSHR* | rs2349415 | T | 0.324 | -0.064 | 0.065 |
| Fasting Glucose | 9q22.32 | *C9orf3* | rs4385527 | G | 0.006 | 0.180 | 0.065 |
| Fasting Glucose | 9q22.32 | *C9orf3* | rs3802457 | G | 0.888 | 0.014 | 0.098 |
| Fasting Glucose | 9q33.3 | *DENND1A* | rs10818854 | G | 0.815 | 0.019 | 0.083 |
| Fasting Glucose | 9q33.3 | *DENND1A* | rs2479106 | G | 0.544 | -0.035 | 0.057 |
| Fasting Glucose | 9q33.3 | *DENND1A* | rs10986105 | C | 0.721 | -0.030 | 0.085 |
| Fasting Glucose | 11q22.1 | *YAP1* | rs1894116 | G | 0.065 | -0.106 | 0.058 |
| Fasting Glucose | 12q13.2 | *RAB5B/SUOX* | rs705702 | G | 0.821 | -0.012 | 0.054 |
| Fasting Glucose | 12q14.3 | *HMGA2* | rs2272046 | C | 0.728 | 0.047 | 0.136 |
| Fasting Glucose | 16q12.1 | *TOX3* | rs4784165 | T | 0.268 | -0.054 | 0.049 |
| Fasting Glucose | 19p13.3 | *INSR* | rs2059807 | G | 0.657 | -0.023 | 0.052 |
| Fasting Glucose | 20q13.2 | *SUMO1P1* | rs6022786 | G | 0.342 | -0.049 | 0.052 |
| Fasting Insulin | 2p21 | *THADA* | rs12468394 | C | 0.170 | -0.079 | 0.057 |
| Fasting Insulin | 2p21 | *THADA* | rs13429458 | C | 0.403 | 0.059 | 0.071 |
| Fasting Insulin | 2p21 | *THADA* | rs12478601 | T | 0.135 | 0.083 | 0.056 |
| Fasting Insulin | 2p16.3 | *LHCGR* | rs13405728 | G | 0.373 | 0.055 | 0.062 |
| Fasting Insulin | 2p16.3 | *FSHR* | rs2268361 | T | 0.563 | -0.027 | 0.048 |
| Fasting Insulin | 2p16.3 | *FSHR* | rs2349415 | T | 0.648 | 0.030 | 0.065 |
| Fasting Insulin | 9q22.32 | *C9orf3* | rs4385527 | G | 0.740 | 0.022 | 0.066 |
| Fasting Insulin | 9q22.32 | *C9orf3* | rs3802457 | G | 0.769 | -0.029 | 0.099 |
| Fasting Insulin | 9q33.3 | *DENND1A* | rs10818854 | G | 0.707 | 0.031 | 0.083 |
| Fasting Insulin | 9q33.3 | *DENND1A* | rs2479106 | G | 0.734 | -0.020 | 0.058 |
| Fasting Insulin | 9q33.3 | *DENND1A* | rs10986105 | C | 0.813 | -0.020 | 0.087 |
| Fasting Insulin | 11q22.1 | *YAP1* | rs1894116 | G | 0.990 | -0.001 | 0.058 |
| Fasting Insulin | 12q13.2 | *RAB5B/SUOX* | rs705702 | G | 0.382 | -0.047 | 0.054 |
| Fasting Insulin | 12q14.3 | *HMGA2* | rs2272046 | C | 0.397 | -0.119 | 0.141 |
| Fasting Insulin | 16q12.1 | *TOX3* | rs4784165 | T | 0.721 | -0.019 | 0.054 |
| Fasting Insulin | 19p13.3 | *INSR* | rs2059807 | G | 0.735 | 0.018 | 0.053 |
| Fasting Insulin | 20q13.2 | *SUMO1P1* | rs6022786 | G | 0.436 | -0.041 | 0.053 |
| 2h-Glucose | 2p21 | *THADA* | rs12468394 | C | 0.773 | 0.016 | 0.057 |
| 2h-Glucose | 2p21 | *THADA* | rs13429458 | C | 0.634 | -0.034 | 0.071 |
| 2h-Glucose | 2p21 | *THADA* | rs12478601 | T | 0.564 | -0.032 | 0.055 |
| 2h-Glucose | 2p16.3 | *LHCGR* | rs13405728 | G | 0.259 | 0.070 | 0.062 |
| 2h-Glucose | 2p16.3 | *FSHR* | rs2268361 | T | 0.135 | 0.070 | 0.047 |
| 2h-Glucose | 2p16.3 | *FSHR* | rs2349415 | T | 0.348 | -0.061 | 0.065 |
| 2h-Glucose | 9q22.32 | *C9orf3* | rs4385527 | G | 0.514 | -0.043 | 0.065 |
| 2h-Glucose | 9q22.32 | *C9orf3* | rs3802457 | G | 0.686 | -0.040 | 0.098 |
| 2h-Glucose | 9q33.3 | *DENND1A* | rs10818854 | G | 0.279 | 0.089 | 0.083 |
| 2h-Glucose | 9q33.3 | *DENND1A* | rs2479106 | G | 0.194 | -0.074 | 0.057 |
| 2h-Glucose | 9q33.3 | *DENND1A* | rs10986105 | C | 0.152 | -0.123 | 0.086 |
| 2h-Glucose | 11q22.1 | *YAP1* | rs1894116 | G | 0.022 | -0.132 | 0.058 |
| 2h-Glucose | 12q13.2 | *RAB5B/SUOX* | rs705702 | G | 0.980 | 0.001 | 0.054 |
| 2h-Glucose | 12q14.3 | *HMGA2* | rs2272046 | C | 0.718 | -0.049 | 0.136 |
| 2h-Glucose | 16q12.1 | *TOX3* | rs4784165 | T | 0.098 | -0.086 | 0.052 |
| 2h-Glucose | 19p13.3 | *INSR* | rs2059807 | G | 0.076 | -0.092 | 0.052 |
| 2h-Glucose | 20q13.2 | *SUMO1P1* | rs6022786 | G | 0.847 | -0.010 | 0.052 |
| 2h-Insulin | 2p21 | *THADA* | rs12468394 | C | 0.816 | -0.013 | 0.054 |
| 2h-Insulin | 2p21 | *THADA* | rs13429458 | C | 0.085 | 0.115 | 0.067 |
| 2h-Insulin | 2p21 | *THADA* | rs12478601 | T | 0.438 | 0.041 | 0.053 |
| 2h-Insulin | 2p16.3 | *LHCGR* | rs13405728 | G | 0.327 | 0.058 | 0.059 |
| 2h-Insulin | 2p16.3 | *FSHR* | rs2268361 | T | 0.512 | 0.029 | 0.045 |
| 2h-Insulin | 2p16.3 | *FSHR* | rs2349415 | T | 0.088 | -0.103 | 0.060 |
| 2h-Insulin | 9q22.32 | *C9orf3* | rs4385527 | G | 0.145 | -0.091 | 0.063 |
| 2h-Insulin | 9q22.32 | *C9orf3* | rs3802457 | G | 0.534 | -0.059 | 0.094 |
| 2h-Insulin | 9q33.3 | *DENND1A* | rs10818854 | G | 0.721 | 0.028 | 0.079 |
| 2h-Insulin | 9q33.3 | *DENND1A* | rs2479106 | G | 0.337 | -0.052 | 0.055 |
| 2h-Insulin | 9q33.3 | *DENND1A* | rs10986105 | C | 0.263 | -0.090 | 0.081 |
| 2h-Insulin | 11q22.1 | *YAP1* | rs1894116 | G | 0.967 | 0.002 | 0.055 |
| 2h-Insulin | 12q13.2 | *RAB5B/SUOX* | rs705702 | G | 0.485 | -0.036 | 0.052 |
| 2h-Insulin | 12q14.3 | *HMGA2* | rs2272046 | C | 0.446 | -0.096 | 0.125 |
| 2h-Insulin | 16q12.1 | *TOX3* | rs4784165 | T | 0.693 | -0.020 | 0.050 |
| 2h-Insulin | 19p13.3 | *INSR* | rs2059807 | G | 0.353 | -0.046 | 0.050 |
| 2h-Insulin | 20q13.2 | *SUMO1P1* | rs6022786 | G | 0.690 | -0.019 | 0.048 |
| HOMA-IR | 2p21 | *THADA* | rs12468394 | C | 0.211 | -0.071 | 0.057 |
| HOMA-IR | 2p21 | *THADA* | rs13429458 | C | 0.526 | 0.044 | 0.070 |
| HOMA-IR | 2p21 | *THADA* | rs12478601 | T | 0.191 | 0.072 | 0.055 |
| HOMA-IR | 2p16.3 | *LHCGR* | rs13405728 | G | 0.379 | 0.054 | 0.061 |
| HOMA-IR | 2p16.3 | *FSHR* | rs2268361 | T | 0.812 | -0.011 | 0.047 |
| HOMA-IR | 2p16.3 | *FSHR* | rs2349415 | T | 0.895 | 0.008 | 0.065 |
| HOMA-IR | 9q22.32 | *C9orf3* | rs4385527 | G | 0.457 | 0.048 | 0.065 |
| HOMA-IR | 9q22.32 | *C9orf3* | rs3802457 | G | 0.809 | -0.024 | 0.098 |
| HOMA-IR | 9q33.3 | *DENND1A* | rs10818854 | G | 0.672 | 0.035 | 0.082 |
| HOMA-IR | 9q33.3 | *DENND1A* | rs2479106 | G | 0.656 | -0.025 | 0.057 |
| HOMA-IR | 9q33.3 | *DENND1A* | rs10986105 | C | 0.760 | -0.026 | 0.085 |
| HOMA-IR | 11q22.1 | *YAP1* | rs1894116 | G | 0.857 | -0.010 | 0.057 |
| HOMA-IR | 12q13.2 | *RAB5B/SUOX* | rs705702 | G | 0.591 | -0.029 | 0.053 |
| HOMA-IR | 12q14.3 | *HMGA2* | rs2272046 | C | 0.473 | -0.099 | 0.138 |
| HOMA-IR | 16q12.1 | *TOX3* | rs4784165 | T | 0.717 | -0.019 | 0.054 |
| HOMA-IR | 19p13.3 | *INSR* | rs2059807 | G | 0.733 | 0.018 | 0.052 |
| HOMA-IR | 20q13.2 | *SUMO1P1* | rs6022786 | G | 0.301 | -0.054 | 0.052 |
